# Guidewire‐assisted successful closure of mis‐punctured transverse colon during endoscopic ultrasound‐guided cyst drainage

**DOI:** 10.1002/deo2.70112

**Published:** 2025-04-11

**Authors:** Naoki Fujita, Hideki Kamada, Ryota Nakabayashi, Takuma Yamashita, Shima Mimura, Hiroki Yamana, Kiyoyuki Kobayashi, Minoru Oshima, Keiichi Okano, Hideki Kobara

**Affiliations:** ^1^ Department of Gastroenterology and Neurology Faculty of Medicine Kagawa University Kagawa Japan; ^2^ Sanuki Municipal Hospital Kagawa Japan; ^3^ Division of Innovative Medicine for Hepatobiliary and Pancreatology Faculty of Medicine, Kagawa University Kagawa Japan; ^4^ Department of Gastroenterological Surgery Faculty of Medicine, Kagawa University Kagawa Japan

**Keywords:** complications, drainage, endoscopic ultrasound‐guided fine needle aspiration, punctures, transverse colon

## Abstract

Endoscopic ultrasound‐guided cyst drainage (EUS‐CD) was performed for walled‐off pancreatic necrosis. Computed tomography performed the next day showed that the tip of the external drainage tube was located in the transverse colon, confirming an accidental EUS‐CD‐associated complication of mis‐puncture. A colonoscopy was performed to confirm the puncture site, which was identified as a defect measuring 2 mm in diameter. The external drainage tube was removed immediately via a guidewire. We intentionally retained the guidewire as a landmark for the puncture site in the colon. The defect was approximated using an endoclip under full vision, with the guidewire covered with surrounding mucosa. After removing the guidewire smoothly, complete closure was achieved with additional endoclips. Four days later, EUS‐CD was successfully repeated, resulting in the resolution of the walled‐off pancreatic necrosis. This is the first known case in which an accidental puncture of the transverse colon occurred during EUS‐CD for walled‐off pancreatic necrosis, and guidewire‐guided clip closure of the colon contributed to troubleshooting.

## INTRODUCTION

Endoscopic ultrasound‐guided cyst drainage (EUS‐CD) is widely used for the treatment of walled‐off pancreatic necrosis (WON) following acute pancreatitis. To perform EUS‐CD safely, it is important to understand how to avoid and effectively manage complications. Early complications of EUS‐CD include bleeding and perforation, and late complications include worsening infections. However, there are few reports of accidental puncture of adjacent organs during EUS‐CD and of successful closure for this mis‐puncture. We report a case of guidewire‐assisted successful closure of a mis‐punctured transverse colon during EUS‐CD.

## CASE REPORT

**FIGURE 1:**
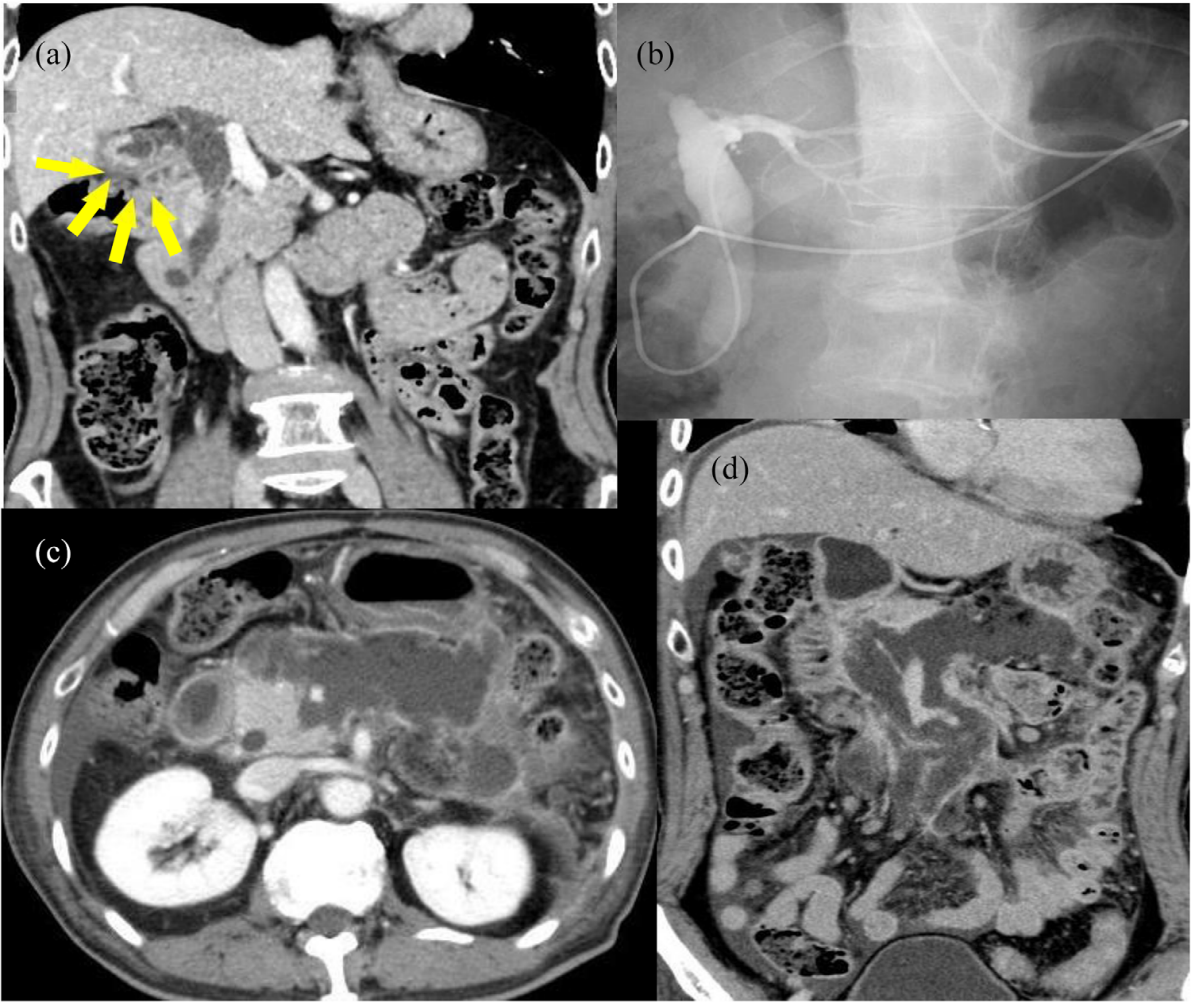
(a) An irregular wall thickening with contrast effect is visible, and gallbladder cancer was suspected. (b) ERCP was performed by the previous physician, and an ENBD tube was placed in the bile duct. (c and d) The patient developed PEP. Additionally, a post‐pancreatitis pseudocyst formed that extended from the peripancreatic area to the lower pole of the left kidney. ERCP, endoscopic retrograde cholangiopancreatography; ENBD, endoscopic nasobiliary drainage; PEP, post‐ERCP pancreatitis.

**FIGURE 2:**
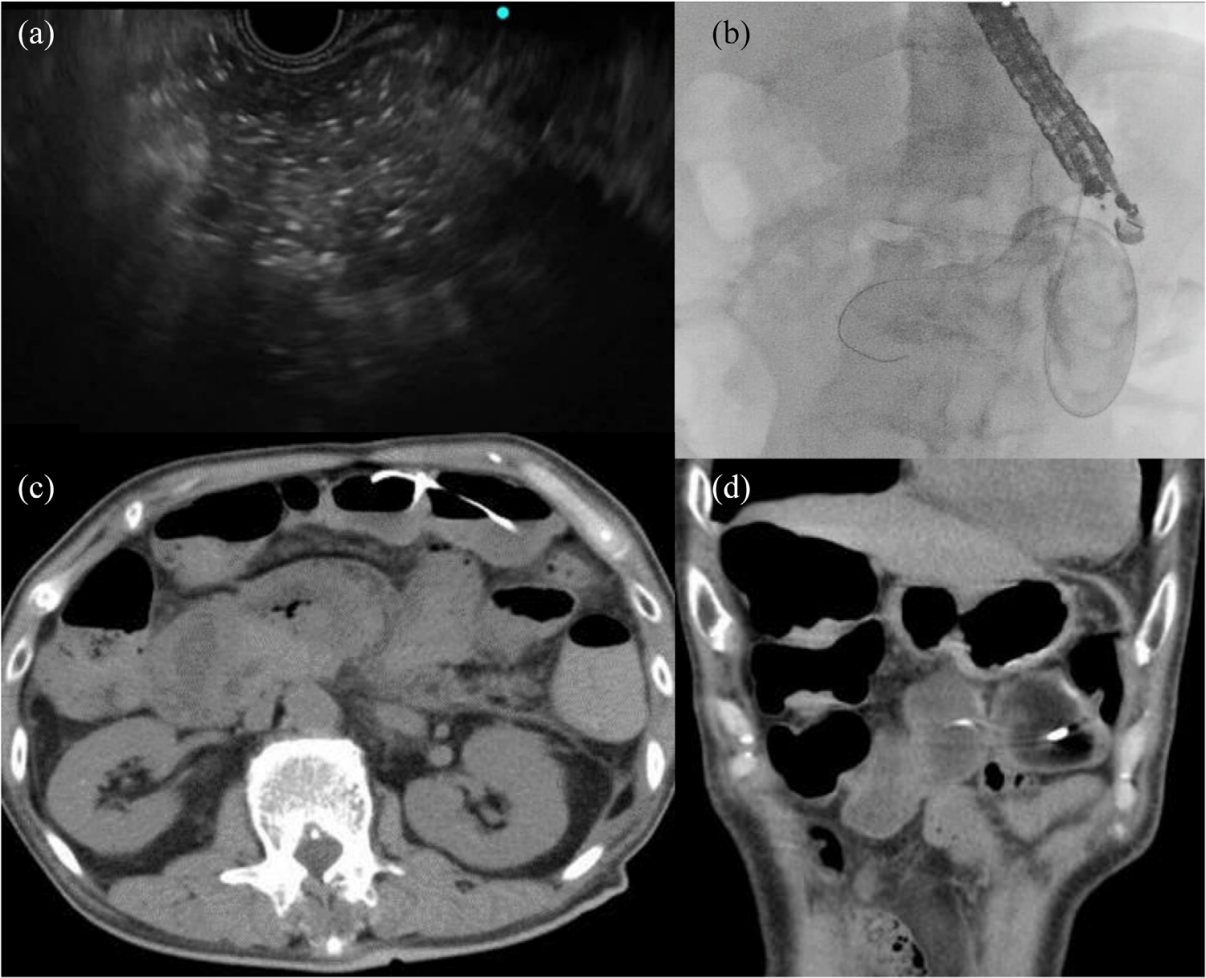
(a–d) EUS‐CD. The cyst (the lumen of the colon was thought to be the cyst) was punctured, and an ENBD tube was placed. The next day, CT showed that the ENBD tube had been placed in the transverse colon, which was an accidental puncture. EUS‐CD, endoscopic ultrasound‐guided cyst drainage; ENBD, endoscopic nasobiliary drainage; CT, computed tomography.

**FIGURE 3:**
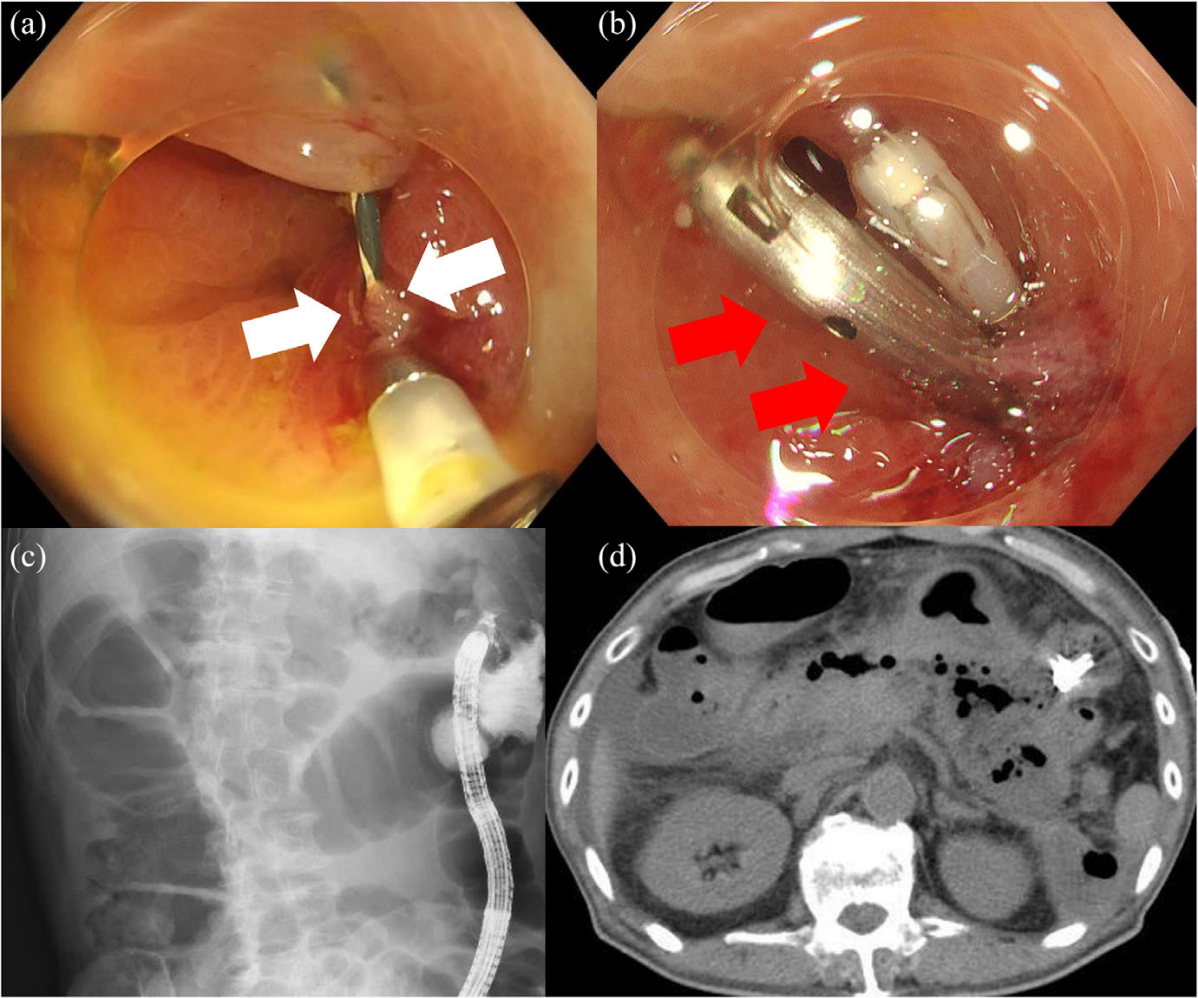
(a) The defect was approximated using an endoclip under full vision, with the guidewire covered with surrounding mucosa. A defect measuring 2 mm in diameter is visible (white arrows). (b) Closure was completed successfully. A MANTIS clip (Boston Scientific Japan; red arrows) was placed to increase the strength of the closure. (c) Contrast imaging confirmed no leakage of contrast media outside the intestinal tract. (d) CT performed the next day showed no evidence of free air or peritonitis. CT, computed tomography.

**FIGURE 4:**
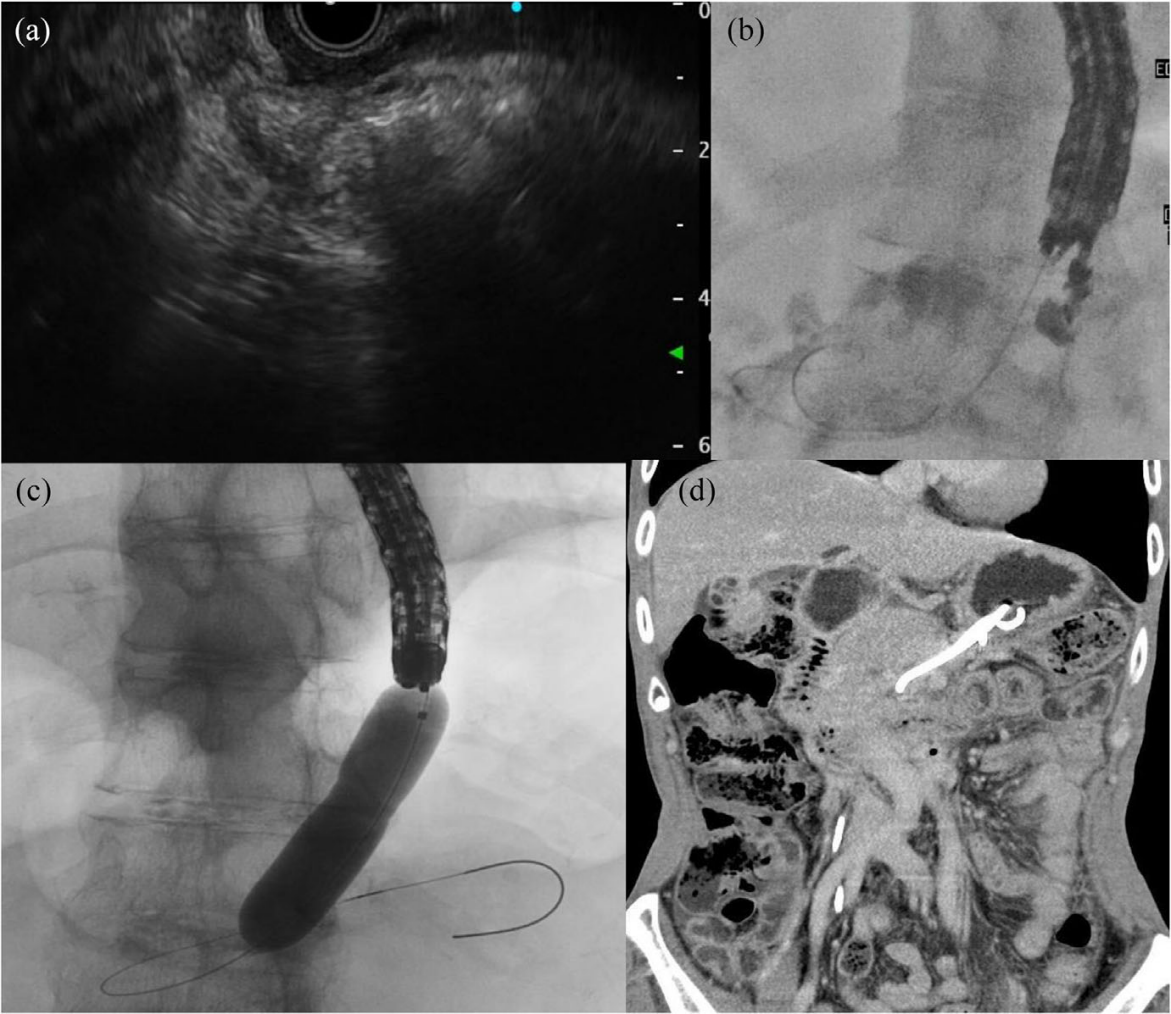
(a and b) Repeat EUS‐CD confirmed that the ENBD tube was successfully placed in the WON. EUS‐CD, endoscopic ultrasound‐guided cyst drainage; ENBD, endoscopic nasobiliary drainage; WON, walled‐off pancreatic necrosis. (c) Necrosectomy was performed by dilating the fistula with a biliary dilatation balloon. (d) This is after the necrosectomy is completed.

A 71‐year‐old man presented to his previous hospital for further examination of a thickened gallbladder wall noted on ultrasonography. Computed tomography (CT), magnetic resonance imaging, abdominal ultrasonography, and EUS strongly suggest gallbladder cancer. Endoscopic retrograde cholangiopancreatography (ERCP) was performed to obtain a pathologic diagnosis, and an endoscopic nasobiliary drainage (ENBD) tube was placed in the bile duct. In retrospect, ERCP may have been unnecessary because it is difficult to diagnose gallbladder cancer using preoperative pathology. However, ERCP was performed by a previous physician, and the details were unknown. Cytology results suggested adenocarcinoma. However, the patient developed post‐ERCP pancreatitis, classified as severe in accordance with the 2021 Japanese guidelines for the management of acute pancreatitis. The patient continued conservative treatment for post‐ERCP pancreatitis, but 2 weeks later, he developed a fever of >38°C and elevated inflammatory markers. Contrast‐enhanced CT revealed multiple fluid collections extending from the peripancreatic area to the left anterior pararenal space. The patient was then referred to our hospital for treatment of infected necrotic collections. He was treated with antibiotics for 2 weeks, during which time, the C‐reactive protein level decreased to 50 mg/L, but he continued to have a fever of >38°C (Figure 1). CT revealed WON with internal gas images extending from the peripancreas to the lower pole of the left kidney. Because lumen‐apposing metal stents (LAMS) were unavailable, EUS‐CD was performed to decompress the WON. A cystic lesion containing air was identified and punctured transgastrically with a 19‐gauge needle. A 0.025‐inch guidewire was advanced into the cystic lesion, and contrast was injected to confirm a shadow defect that appeared to be necrotic tissue. Finally, a drainage tube (nasobiliary pancreatic drainage set, 6‐Fr pigtail type; Cook Medical Japan) was placed in the cystic lesion. The next day, the patient complained of left upper abdominal pain. Therefore, CT was performed to confirm the location of the tube, which was identified in the transverse colon (Figure 2). Immediate removal of the drainage tube was planned. First, a colonoscopy was performed to confirm the puncture site. No pretreatment for colonoscopy was performed as the patient had fasted since the previous day's procedure. A defect in the transverse colon measuring 2 mm in diameter was identified endoscopically. A 0.025‐inch guidewire (VisiGlide 2; Olympus) was inserted into the drainage tube, and the tube was removed via the guidewire. We intentionally retained the guidewire as a landmark indicating the puncture site in the colon. The defect was approximated using an endoclip (HX‐610‐090SC; Olympus) under full vision, with the guidewire covered with surrounding mucosa. After removing the guidewire smoothly, complete closure was achieved with additional endoclips. Clips specialized for closure (MANTIS; Boston Scientific Japan) were used to maintain tensile strength. The procedure was terminated after confirming the lack of contrast medium leakage into the peritoneal cavity. Additionally, the risk of bacterial leakage from the gastric perforation and associated severe peritonitis was considered low. Therefore, the patient continued to fast and was treated with antibiotics. CT performed the next day showed no signs of peritonitis or other complications (Figure 3). Four days later, after careful observation, EUS‐CD was repeated, and a drainage tube was placed correctly in the WON (Figure 4). An endoscopic necrosectomy was performed subsequently, and the WON disappeared. In this case, a necrosectomy was performed by dilating the fistula with a biliary dilatation balloon (CRE PRO; Boston Scientific Japan) and advancing the endoscope. Fortunately, the WON quickly decreased in size, and the necrosectomy was completed in three sessions. Therefore, a LAMS was not placed. The patient's general condition improved, and surgery was performed subsequently for gallbladder cancer (Figure 4).

## DISCUSSION

WON with infection requires appropriate drainage. EUS‐CD is becoming increasingly popular owing to its minimally invasive nature.^1^ The technical success rate of EUS‐CD is reported to be >90%, and the clinical success rate is 79%–85%. Early complications include bleeding and stent migration, while late complications include worsening cyst infection.^2^, ^3^, ^4^, ^5^ The incidence of these complications has been reported to range from 1.5% to 26.2%.^2^, ^3^, ^4^, ^5^ However, reports of punctures of other organs are rare, with only one reported case of gallbladder puncture.^6^ In the report, the authors noted that the patient could not be placed in the supine position. Additionally, the presence of a large amount of biliary sludge made it difficult to differentiate the gallbladder from an inflammatory cyst, which led to an accidental gallbladder puncture. In our case, the patient had severe constipation with a large amount of fecal matter containing air in the colon. These issues made it difficult to distinguish WON from the air‐containing inflammatory regions observed on EUS, leading to accidental colonic puncture followed by tube deployment. Additionally, our patient complained of abdominal pain the day after the procedure, and CT immediately confirmed a mis‐puncture. The tube was removed immediately, and the puncture site could be closed endoscopically because the tissue around the site was not indurated. The usefulness of endoscopic clipping^7^, ^8^ and over‐the‐scope clips (Ovesco Endoscopy) for the closure of sigmoid colon perforation by biliary stents (7‐Fr) has been reported.^9^ In our case, owing to the short interval from the puncture and the small 6‐Fr tube, we chose standard endoclips. We determined that if the tube was left in place, we would be unable to successfully close the puncture site. We also considered the possibility that the endoclips we used would make it difficult to remove the tube. However, we believed that without a landmark, we would lose sight of the puncture site; therefore, we decided to retain the guidewire. As reported, even with a marking clip nearby,^9^ precise identification of small puncture sites is challenging; therefore, we used the guidewire as a landmark during clipping closure. For smooth guidewire removal, care was taken not to bite the guidewire directly, and clipping was done to wrap the guidewire with the surrounding mucosa. A MANTIS clip (Boston Scientific Japan) with a specific claw was efficacious in adding extra strength to the closure site. The patient's condition remained stable, and CT performed the day after the procedure revealed no evidence of perforation.

In this case, early intervention for the accidental puncture allowed endoscopic management. However, in retrospect, the WON had inconsistent echo‐luminance and unclear boundaries on EUS images. Additionally, the contrast spread was not uniform on fluoroscopic images. To prevent accidental punctures, careful observation of the target and sufficient confirmation of contrast spread post‐puncture are essential. Additionally, LAMS has recently become popular; if a similar case were to occur with LAMS, the puncture site would likely be large and surgery would be required (Supporting information).

## CONFLICT OF INTEREST STATEMENT

Co‐author Hideki Kobara is the Associate Editor of *DEN Open*.

## ETHICS STATEMENT

N/A

## PATIENT CONSENT STATEMENT

Informed consent for this case report was obtained from the patient.

## Supporting information



Supporting in formation
